# The combined effect of pit and fissure sealant application and oral health education on oral health status of children aged 6–9 years: a 12-month follow-up study in Northeast China

**DOI:** 10.1186/s12903-023-03467-0

**Published:** 2023-10-27

**Authors:** Liwen Chen, Ming Wu, Qing Gao, Siyu Zhang, Kaiqiang Zhang, Jian Li, Chang Cha, Xiaoli Li, Lu Liu

**Affiliations:** 1 Department of Preventive Dentistry, School and Hospital of Stomatology, Liaoning Provincial Key Laboratory of Oral Diseases, China Medical University, Shenyang, 110101 China; 2 Liaoning Center for Disease Prevention And Control, Shenyang, 110172 China

**Keywords:** Oral health, Factors, Dental caries, Pit and fissure sealant

## Abstract

**Background:**

Children aged 6–9 years are vulnerable to dental caries due to age-related limitations and a lack of adequate knowledge regarding oral health and hygiene practices. This study aimed to establish a cohort of children aged between 6 and 9 years and conducted a 12-month follow-up to examine the combined effect of pit and fissure sealant (PFS) application and oral health education on their oral health status.

**Methods:**

A cohort study with 12-month follow-up was conducted in Liaoning province, China. A multi-stage stratified cluster sampling approach was employed in the study. The enrolled 6- to 9-year-old children were all from the selected primary schools, who had resided in the designated area for at least 6 months. Children who were unable to cooperate with the examiner or without informed consent from their guardians were excluded. Experienced dental professionals examined the oral health status of primary school children aged 6–9 years. All children and their guardians were mandated to complete a questionnaire (qualitative data) at the beginning of the study. In the experiment group, children underwent PFS application and chairside oral health education. Clinical examinations and questionnaire surveys were repeated at the 12-month follow-up. The chi-square test and binary logistic regression were conducted to investigate the potential risk factors associated with dental caries prevalence (dependent variable). Independent variables were items from the questionnaire (such as living place, parents’ education level and children’ birth weight). The significant variables identified in the chi-square tests were subsequently included in the binary logistic regression analysis.

**Results:**

A total of 4,085 children aged 6–9 years were included in the study, with 1805 participants assigned to the experiment group and 2280 to the control group. At baseline, the caries rates of the experimental and control group were 77.95% and 80.35%, respectively without any statistically significant differences. However, at the 12-month follow-up, the caries rate in the experimental group (83.65%) was significantly lower than that in the control group (86.62%) (*P* < 0.05). The results from the binary logistic regression analysis indicated that parents with a college degree and children in the experimental group exhibited lower caries rates. Conversely, higher caries rates were associated with the consumption of sweet beverages and foods more than once a day and a lack of knowledge regarding the causes of caries (*P* < 0.05).

**Conclusions:**

In Liaoning, China, children aged 6 to 9 years exhibited a high prevalence of dental caries. Several factors, including the parent’s education level, the frequency of consuming sweet beverages and foods, and the children’s understanding of the cause of caries, significantly affected the caries prevalence rates. The implementation of PFS application and oral health education effectively reduce the caries rate among the surveyed children.

## Background

Dental caries is a complex and multifactorial disease with a worldwide prevalence. It is characterised by localised progressive destruction of susceptible dental hard tissues, resulting from long-term interactions between acidogenic bacteria, fermentable carbohydrates, and host factors [1]. Being one of the most prevalent oral diseases [2], it affects individuals from all age groups throughout their lives. Children aged 6–9 years are particularly vulnerable to dental caries due to their stage of caries susceptibility as their first permanent molars (FPM) typically erupt by 6 years of age. During this period, children are more susceptible to dental caries due to age-related limitations and a lack of adequate knowledge regarding oral health and hygiene practices. If left untreated, dental caries can lead to significant morbidity, including pain, infection, and school absenteeism, adversely affecting students’ physical and psychological well-being [3]. The financial burden of treating oral diseases is substantial for families and national healthcare systems, particularly in developing countries like China. This burden further contributes to the disparities between economically developed and developing nations [4]. National oral health surveys have been conducted to monitor the current trends in the prevalence of oral diseases and assist in making appropriate public health policies to reduce their prevalence. Over the past few decades, four national oral health surveys have been conducted in China. According to the latest findings from an epidemiological survey assessing the oral health status of residents in Liaoning, 76.3% of children aged 3–5 years exhibited primary dental caries, and 51.1% of 12-year-old children had dental caries in their permanent teeth [5]. These findings are relatively higher compared with the prevalence rates reported in the Fourth National Oral Health Survey, which were 70.9% and 34.5%, respectively [6]. These data indicate an increasing prevalence of dental caries in children with the development of socioeconomic status and changes in dietary habits.

Despite the four national oral health surveys conducted in the past few decades, there remains a lack of data on the 6- to 9-year age group [7]. This age range holds significant importance in children’s lives as it encompasses the transitional phase from primary dentition to permanent teeth, during which both dentitions are susceptible to dental caries. Among the teeth affected by caries, the FPM holds considerable prominence in children [8]. The FPM, due to its stable position after eruption, plays a crucial role in guiding occlusal development, stabilising the dentition, and maintaining normal masticatory function and orofacial harmony [8, 9]. Pit and fissure sealants (PFS) have been employed to prevent caries in the FPM and have demonstrated effective outcomes [10].

Since 2008, the central government has implemented a comprehensive intervention program targeting children’s oral diseases in the central and western regions. This program offers free oral health education, clinical examinations, and PFS application on FPM for children aged 6 to 9 years [11]. However, initially, Liaoning was not included in this project due to its location. Occupying 1.5% of China’s land area, Liaoning is situated in Northeast China, serving as a significant hub for the economy, politics, and culture of the region, connecting the Yellow Sea Economic Zone and the Northeast Economic Zone. Therefore, it is crucial to pay special attention to the oral health status of children in Liaoning. Eventually, in 2014, Liaoning was included in the nation’s comprehensive intervention program. However, the allocated budget from the national intervention program for Liaoning only covered the treatment of 15,000 teeth, which was far from meeting the needs of the province’s 300,000 children. Consequently, in 2018, Liaoning initiated its provincial program to bridge this gap.

Currently, limited research exists on the combined effect of PFS and oral health education on the oral health status of 6- to 9-year-old children, as evidenced by the lack of relevant studies found in the searched databases. Hence, this study aimed to establish a cohort of 6- to 9-year-old children and conduct a 12-month follow-up to assess their oral health status in Liaoning province through oral examinations and questionnaires. Additionally, the combined effect of PFS and oral health education on these children was analysed. Furthermore, the associated risk factors were investigated.

## Materials and methods

### Ethical clearance

This study was approved by the Ethics Committee of the School of Stomatology, China Medical University (approval number: k2022032).

### Inclusion and exclusion criteria

The inclusion criteria for this study were as follows: children aged between 6 and 9 years, enrolled in the selected primary schools, residing in the designated area for a minimum of 6 months, with informed consent provided by their guardians, and those capable of cooperating during the examination.

The exclusion criteria for this study were as follows: children who were unable to cooperate with the examiner, those without informed consent from their guardians, or students with psychiatric disorders.

### Sample selection

**Fig. 1:**
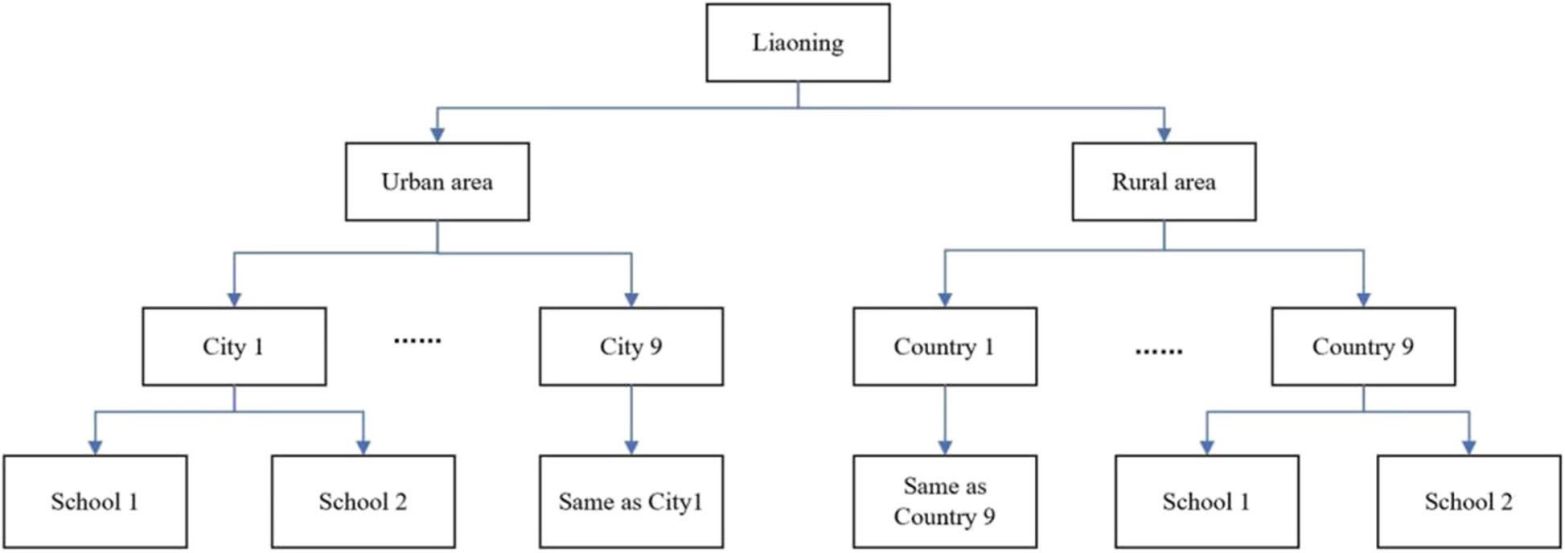
Flowchart representing the sample selection

The study employed a multi-stage stratified cluster sampling approach. In the first stage, nine districts within Liaoning were chosen for inclusion. In the second stage, one city and one county were randomly selected from the nine districts using probability proportional to size sampling. In the third and fourth stages, one or two schools were randomly chosen from each city (county), and from these schools, children aged 6–9 years were selected using cluster sampling (Fig. 1).

The sample size 446 for this study was determined using the following formula: n = deff(u_α/2_^2^p(1 − p)/δ^2^). A design effect (deff = 4.5), a significance level (α = 0.05), a margin of error (δ = 10%), and a non-response rate (20%) were included. The caries prevalence rate of 70.9% was adopted based on data from the Fourth National Oral Health Survey [6].

### Questionnaire survey

All children and their guardians were required to complete a questionnaire at the baseline and the follow-up stage. Before the survey, the questionnaire interviewers underwent training to ensure a high level of agreement in terms of the questionnaire answers. The interviewers explained the questions and items to the children to help them better understand the children’s version of the questionnaire. Subsequently, the children independently completed the questionnaire. The same procedure was followed for the children’s guardians, aiding them in completing the guardians’ version of the questionnaire. The questionnaire comprised the following sections:

1. The respondent’s identification and their relationship to the child.
2. School’s name and the examination date.
3. Socio-demographic background of the children, including age, height, weight, etc.
4. Early life factors, such as birth weight and feeding approaches within the first 6 months of life.
5. Oral health-related behaviour, including sugar consumption, tooth-brushing behaviour, usage of fluoride paste, etc.
6. Oral health-related knowledge and attitudes, such as understanding the cause of dental caries, recognising the necessity of pit and fissure sealant usage, emphasising the importance of protecting the FPM, etc.
7. In guardians’ version of the questionnaire included three questions associated with oral health-related habits, specifically focusing on topics such as the reasons for dental visits and the reasons for irregular dental visits.

### Clinical examination

Each child underwent a clinical examination using the World Health Organisation (WHO)-recommended methods, which involved the use of a plane mouth mirror and a Community Periodontal Index probe under an artificial light source. Dental caries were diagnosed and scored based on the WHO standard criteria [12]. Following the oral health education provided by experienced dental staff, the enrolled children underwent their first examination and were subsequently divided into two groups. Children whose guardians consented to PFS application on their FPM were assigned to the experimental group, with informed consent forms signed and maintained for records. The remaining children were assigned to the control group. PFS application was thoroughly performed on the FPM of the children belonging to the experimental group, followed by chairside oral health education. The children belonging to the control group only underwent clinical examinations. After a 12-month follow-up, the enrolled children underwent another clinical examination.

### Quality control

All examiners possessed qualifications as oral practitioners and had more than 3 years of clinical work experience. The recorders comprised dentists or nurses who also had the necessary qualifications and work experience. Prior to the examination, theoretical and clinical training sessions were conducted to ensure consistent and reliable results in terms of inter-examiner and intra-examiner reproducibility (kappa > 0.85). A standard examiner was appointed, and duplicate examinations were randomly performed on 5% of the children to ensure data reliability.

### Data analysis

Data collection was performed using EpiData software (www.epidata.dk). Each checklist or questionnaire was checked twice to avoid input errors and ensure the accuracy and objectivity of data entry.

Data analysis was performed using IBM SPSS statistics software (version 20.0). Statistical significance was set at *P* < 0.05. Descriptive analyses were performed to present the oral health status using the DMFT index and caries prevalence. The chi-square test was used to analyse the prevalence of dental caries across different age groups and selected variables. Additionally, it was employed to examine the sex and urban/rural distribution differences between the experimental and control groups. Binary logistic regression was employed to investigate the relationships between dental caries prevalence (dependent variable) and the significant variables identified in the chi-square test. Statistical significance was set at 0.05 for all tests. Cohen’s kappa statistic values were used to evaluate inter-examiner and intra-examiner reproducibilities of the clinical examination.

## Results

### Distribution of the study population

This study ultimately included 4085 children aged 6–9 including 1805 children in the experimental group and 2280 children in the control group. A total of 296 participants were not included in the study because they did not complete the questionnaire survey during the follow-up phase.

**Fig. 2:**
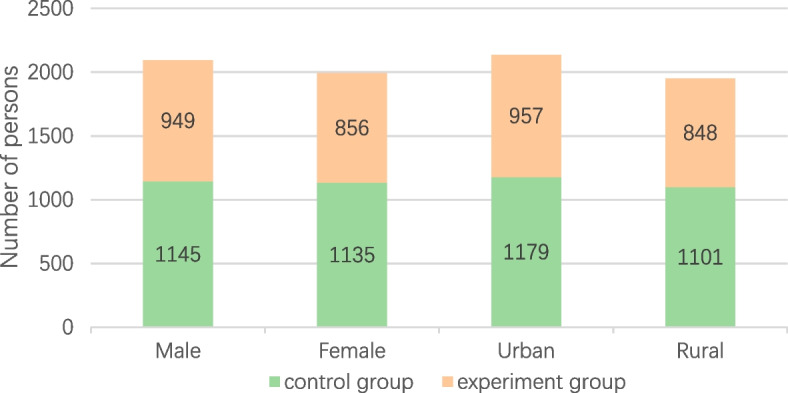
Distribution of the study population

There were no significant differences in sex and urban/rural distribution between the two groups at baseline (*P* > 0.05). The study’s experimental and control groups showed a fairly balanced representation of both genders, with a slight majority of boys, and a slightly higher proportion of children from urban areas compared to rural areas in both groups (Fig. 2).

### Prevalence of dental caries in the study population

**Fig. 3:**
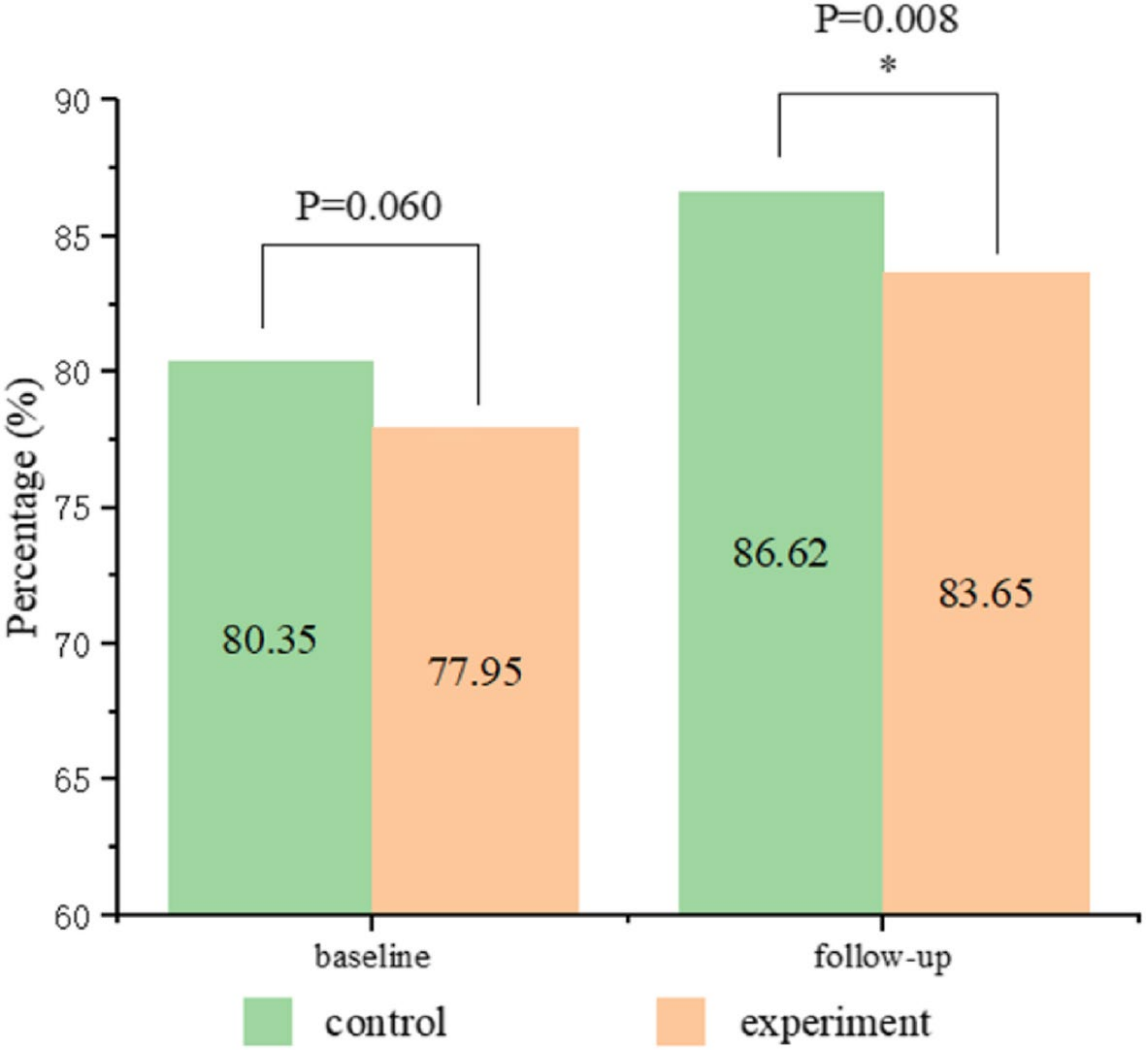
Distribution of the study population

At baseline, the dental caries prevalence rates of the experimental and control groups were 77.95% and 80.35%, respectively. No statistical differences (*P = 0.06 > *0.05) were observed between the two groups at baseline. However, after the 12-month follow-up, the incidence of dental caries in the control group (86.62%) was higher than that in the experimental group (83.65%), which was statistically significant (*P = 0.008* < 0.05) (Fig. 3).

### Characteristics of behaviours and knowledge of oral health

#### Knowledge and attitude toward oral health

Eight questions in the children’s version of the questionnaire and nineteen questions in the guardians’ version were used to assess their knowledge and attitudes toward oral health. The data revealed a significant improvement in the accuracy rates of these questions for children and their guardians (*P* < 0.05) after oral health education (Table 1).

**Table 1:** The number of responses to questions about oral health knowledge and attitudes

	Time	Number of correct or wrong answers		Χ^2^	*P*-value
		Correct	Wrong		
Children	Baseline	20,165	12,515	223.285	0.000
	Follow-up	21,993	10,687		
Guardians	Baseline	35,768	41,847	2066.463	0.000
	Follow-up	44,717	32,898		

#### Oral health-related habits

##### Reasons for dental visit

Regular examinations were the primary reason for dental visits, with 32.89% and 31.05% of children in the experimental and control groups, respectively. In the experimental group, 31.29% of children underwent dental preventive measures and 22.01% underwent treatment for oral diseases, which were the second and third common reasons for dental visits. These accounted for 29.74% and 28.16% in the control group, respectively (Table 2).

**Table 2:** The percentage of responses on reasons for dental visit

	Control group %	Experiment group %
Reasons for dental visit		
Regular examination	31.05	32.89
Dental prevention	29.74	31.29
Dental treatment	28.16	22.01
Unknown reasons	11.05	13.81

##### Reasons for irregular dental visit

In the study, a noticeable proportion of guardians in both groups believed dental visits were not necessary unless there were evident oral health issues. Furthermore, some guardians in the experimental group believed that mild dental decay did not need intervention, and a fair number of guardians in both groups justified infrequent dental visits with the expectation of natural replacement of children’s primary teeth (Table 3).

**Table 3:** The percentage of the response on reasons for irregular dental visit

	Control group %	Experiment group %
Reasons for irregular dental visit		
No oral health problems	19.07	25.30
No severe oral health problems	7.57	10.53
Primary teeth would be replaced	8.61	9.04
Economic problems	4.52	3.46
Inconvenient dental visit	8.01	4.28
Busy with work	7.64	9.30
Children afraid of the dentist	9.74	8.85
No dentist nearby	4.96	3.42
Fear of infectious diseases	8.68	5.95
Distrust of dentist	5.22	4.43
Difficulty in registration	7.34	3.68
Other reasons	8.63	11.76

##### Ways to obtain oral health information

In terms of the sources of oral health information, television and videos were the most common sources. A similar proportion (around 15%) of guardians in both groups obtained knowledge from the dental staff (Table 4).

**Table 4:** The percentage of responses on ways to obtain oral health information

	Control group %	Experiment group %
Ways to obtain oral health information		
Television/videos	15.92	17.42
Newspaper/magazine/popular science books	13.56	15.85
Families/friends	15.32	14.28
Hospital bulletin board	10.82	11.01
Dental staff	15.12	15.02
Community health promotion activities	8.44	7.09
Knowledge obtained from the school by children	14.54	14.07
School for pregnant women	6.29	5.25

### Correlates of dental caries in the study population

We analysed the questionnaire data, including variables such as sex, region, parents’ education, early life factors, oral health-related behaviours and attitudes, and knowledge of oral health to identify correlates of dental caries in the study population. The results of the chi-square test revealed significant associations between dental caries prevalence and region, parents’ education, and birth weight (*P* < 0.05). Specifically, the prevalence of dental caries was higher in children in rural areas compared with those living in urban areas. Children of highly educated parents exhibited a lower risk of dental caries, while children with lower birth weights were more likely to experience caries.

The results further demonstrated significant associations between children’s behaviours and their knowledge of oral health with respect to caries prevalence (*P* < 0.05). It was observed that lower frequencies of sugar consumption, such as ‘sugar-intake less than once per day’ and ‘no sugar-intake before bedtime’ and ‘the initiation of tooth-brushing behaviours at 6 months’, was associated with a decreased caries rate in children. Moreover, children who accurately perceived the importance of various factors, such as ‘PFS being beneficial for teeth’, ‘regular dental visits being necessary’, ‘teeth requiring protection’, and ‘understanding the causes of caries’ exhibited lower levels of caries.

Significant associations were observed between the guardians’ accurate attitudes and knowledge of oral health and lower caries rates in children (*P* < 0.05). The children of guardians who possessed a proper understanding of key aspects such as the preventive effect of PFS on caries and the importance of oral health exhibited lower rates of caries (Table 5).

**Table 5:** Univariate analysis of the potentially significant factors

Factors	N	Number of children with caries	%	Χ^2^	*P*-value
Living place					
City	2136	1765	82.63	25.681	0.000
County	1949	1720	88.25		
Parents’ education					
Junior high school and below	1271	1124	88.43	18.379	0.000
Senior middle school	898	771	85.86		
Bachelor’s degree or above	1916	1590	82.99		
Group					
Control group	2280	1975	86.62	7.074	0.008
Experiment group	1805	1510	83.66		
Birth weight					
< 2.5 kg	466	427	91.63	19.991	0.000
2.5-4.0 kg	3090	2599	84.11		
> 4.0 kg	449	392	87.31		
Unknown	80	67	83.75		
Guardians: Children are born with good or bad teeth, with no correlation with protection					
Yes	707	653	92.36	33.912	0.000
No	3378	2832	83.84		
Guardians: Oral health is important to personal life					
Yes	3547	2976	83.90	42.744	0.000
No	538	509	94.61		
Guardians: It is important to protect children’s first permanent teeth					
Yes	3202	2684	83.82	26.120	0.000
No	882	800	90.70		
Guardians: Deciduous teeth do not require treatment					
Yes	960	862	89.79	20.096	0.000
No	3125	2623	83.94		
Guardians: The use of pit and fissure sealants can prevent caries					
Yes	2967	2491	83.96	15.506	0.000
No	1113	989	88.86		
Guardians: It is normal for gums to bleed while brushing teeth					
Yes	1009	895	88.70	12.409	0.000
No	3071	2585	84.17		
Guardians: Sugar can cause dental caries					
Yes	3004	2517	83.79	20.503	0.000
No	1075	962	89.49		
Guardians: Oral health is important					
Yes	3263	2729	83.63	36.157	0.000
No	820	754	91.95		
Children: Frequency of carbonated drinks (coke/juice) less than once per day					
Yes	3177	2619	82.44	95.734	0.000
No	906	865	95.47		
Children: Frequency of candies/chocolates and other desserts less than once per day					
Yes	2706	2223	82.15	63.934	0.000
No	1379	1262	91.52		
Children: Frequency of sweet milk/yoghurt/milk powder/tea/soybean milk/coffee less than once per day					
Yes	2,636	2,185	82.89	34.711	0.000
No	1,449	1,300	89.72		
Children: No sugar intake before bedtime					
Yes	2,149	1,804	83.95	6.753	0.000
No	1,936	1,681	86.83		
Children: Start brushing teeth at 6 months					
Yes	412	377	91.50	14.02	0.000
No	3,673	3,108	84.62		
Children: The use of pit and fissure sealants is good for teeth					
Yes	3,119	2,639	84.61	5.182	0.023
No	966	846	87.58		
Children: Regular dental visits are necessary					
Yes	3,282	2,778	84.64	6.071	0.048
No	802	706	88.03		
Children: Children are not born with good or bad teeth and they need protection					
Yes	3,689	3,122	84.63	14.131	0.000
No	396	363	91.67		
Children: Know the cause of dental caries					
Yes	796	628	78.89	32.495	0.000
No	3,289	2,857	86.87		

The risk factors identified in the chi-square tests were subsequently included in the binary logistic regression analysis. As presented in Table 6, five factors were significantly associated with children’s caries rate (*P* < 0.05). Specifically, parents with college degrees and children in the experimental group exhibited lower caries rates. Conversely, higher caries rates were associated with the consumption of sweet drinks and foods more than once a day and children’s lack of knowledge regarding the causes of caries (Table 6).

**Table 6:** Binary logistic regression analysis of the prevalence of dental caries

Factors	B	SE	Wald	df	Sig.	Exp(B)	95% CI
Parents’ education							
Junior high school and below			5.681	2	0.058		
Senior middle school	-0.124	0.135	0.854	1	0.355	0.883	0.678–1.150
Bachelor’s degree or above	-0.270	0.116	5.437	1	0.020	0.763	0.608–0.958
Group							
Control group							
Experiment group	-0.253	0.093	7.435	1	0.006	0.776	0.647–0.931
Children: Frequency of carbonated drinks (coke/juice) less than once per day							
Yes							
No	0.912	0.187	23.731	1	< 0.001	2.490	1.725–3.595
Children: Frequency of candies/chocolates and other desserts less than once per day							
Yes							
No	0.430	0.119	13.123	1	< 0.001	1.537	1.218–1.939
Children: Know the cause of dental caries							
Yes							
No	0.410	0.105	15.299	1	< 0.001	1.506	1.227–1.849

## Discussion

The most recent Fourth National Oral Health Survey introduced additional age groups beyond the recommended range by the WHO to observe the trends in oral diseases among school-age children. The new age groups include children aged 3–5 years and 12–15 years [7]. However, it is noteworthy that the crucial age group of 6–9 years is not covered in national surveys or WHO recommendations. This age range is particularly important as it corresponds to the eruption or presence of the FPM in children, which often goes unnoticed by parents, leading to potential decay soon after eruption. Therefore, surveys had been conducted to address the data gap in this age group [13–15]. However, there is a limited significant lack of research on the prevalence of dental caries among children in this age group in Northeast China, with even fewer studies exploring the factors that influence their oral health condition. Therefore, this research aims to present the current oral health status of 6- to 9-year-old children in Liaoning, China.

In this study conducted in Liaoning province, it was found that more than three-fourths of the children surveyed had dental caries. This prevalence rate is considerably higher compared with the rates observed in Hangzhou and Shenzhen, China, among children aged 6–8 years (52.78% and 56.59%, respectively) [15, 16]. Available data from the National Bureau of Statistics [17] indicates that the gross domestic product (GDP) of Shenzhen and Hangzhou exceeds that of the highest GDP city in Liaoning province (3.07 trillion, 1.81 trillion, and 0.72 trillion, respectively). The notable difference in caries prevalence between Liaoning Province and the other two cities might be attributed to variations in economic development levels across the surveyed regions. Higher GDP levels could be associated with increased financial support from the government for managing children’s oral health and improving the socioeconomic status of families [16]. Conversely, lower socioeconomic status has been demonstrated to be significantly associated with a higher risk of dental caries in children and adults, as highlighted in systematic reviews [18, 19]. Moreover, this correlation extends to regional disparities, as observed in studies from China and the Netherlands [16, 20]. Another possible reason might be associated with the earlier initiation of the PFS program in Hangzhou and Shenzhen, with these cities launching city-wide PFS programs in 2010 and 2015, respectively [15]. As a result of years of promoting the PFS program, parents in these cities have increased their knowledge and acceptance of PFS, which has likely influenced the reception rate of PFSs among their children.

This study aimed to examine the combined effect of PFS and oral health education on the oral health status of children aged 6–9 years. A cohort of children was established and followed up for 12 months to assess the outcomes. The follow-up findings revealed a reduction in caries prevalence among the children, indicating the effectiveness of implementing PFS in conjunction with oral health education. These results also indicated significant improvements in oral health knowledge and attitudes among children and their guardians. This aligns with the existing evidence supporting the use of PFS and oral health education as a preventive measure against caries, which has been recommended for over five decades and has proven to be effective [21, 22]. Additionally, previous research has demonstrated the effectiveness of oral health education in improving oral health knowledge and attitudes [23, 24], a finding consistent with the results of this study. A questionnaire survey was employed to assess the attitudes of the children and their guardians towards oral health and their level of healthcare knowledge. The results demonstrated that children and their guardians exhibited enhanced knowledge and more positive attitudes towards oral health at the 12-month follow-up, with a *P*-value < 0.001.

In terms of oral health habits, it was observed that a higher percentage of children in the experimental group visited dentists for regular examinations or dental prevention compared with the control group, while fewer children in the experimental group sought treatment. These findings imply that oral health education led to an improvement in guardians’ awareness of their children’s oral health and the significance of regular dental visits. This is evident from the differences in the reasons for dental visits between the two groups. Regarding the sources of oral health information, approximately 15% of the guardians reported learning from their children, in addition to obtaining knowledge from media sources or dental staff. However, our binary logistic regression analysis revealed that children’s lack of knowledge about the causes of caries was also a risk factor for their caries prevalence. Consequently, in line with previous studies, our findings underscore the importance of focusing on children’s role in promoting oral health knowledge and highlighting the need to consider them as a crucial target group for oral health education [23, 24].

Several variables were found to be associated with caries in children aged 6–9 years in Liaoning province. Notably, children in rural areas exhibited a higher prevalence of caries compared to their urban counterparts, which is consistent with the findings of the Fourth National Oral Health Survey and other national surveys conducted in various countries [25–27]. These results suggest the need for additional preventive dental public health measures targeted towards the rural population. Furthermore, our study revealed a significant association between low birth weight (LBW) and higher caries prevalence in the surveyed children. LBW refers to a newborn weighing ≤ 2.5 kg [28]. While previous studies on the association between LBW and dental caries in children have yielded inconsistent findings [28–30], our study demonstrated a positive association between LBW and caries. This relationship might be attributed to the higher prevalence of developmental defects observed in children with an LBW, as reported in previous studies [28]. However, further investigation is warranted to better understand this association.

The binary logistic regression analysis demonstrated a significant relationship between parent’s education level and children’s caries prevalence. As parents’ education level increased, children’s caries prevalence decreased, which aligns with the findings from similar studies [15, 31]. A study conducted in China provided further insights, revealing that children with highly educated parents were more likely to engage in tooth brushing, brush more frequently, visit the dentist regularly, and undergo regular dental check-ups. Moreover, parents with higher educational backgrounds exhibited a better understanding of PFS and placed greater emphasis on the completion of PFS practices [31]. Furthermore, the results indicated that the combined use of PFS and oral health education was associated with a lower caries prevalence in the experimental group at the 12-month follow-up (*P* < 0.001). Conversely, higher caries rates were associated with the consumption of sweet drinks and foods more than once a day and children’s lack of knowledge about the causes of caries. The association between caries and excessive consumption of sweet food and beverages has been supported by previous studies [32, 33]. A review by Cor van Loveren highlighted that, according to the WHO guideline for caries prevention, reducing the frequency of sugar-containing product consumption is more important and achievable than reducing the amount of sugar in such products [33].

Our study has some limitations. Due to constraints in terms of time, budget, and human resources, certain variables, such as household income, the rate of caries in FPMs, the PFS retention rate, etc., were not included in our study. Moreover, microbial and serotype analysis of cariogenic pathogens in the surveyed children was not conducted in our study, which could be done in future research. Further implementation and investigation of the identified factors and interventions should be promoted in future work.

In conclusion, our findings emphasise the effectiveness of oral health education and the use of PFS in reducing the prevalence of dental caries among children aged 6–9 years in Liaoning, China. Furthermore, parents’ education level, frequency of sweet drinks or food consumption, and children’s knowledge of the causes of caries were identified as significant factors associated with dental caries. These results highlight the importance of collaborative efforts among policymakers, healthcare providers, and educators to can improve oral health outcomes for children in this age group and address existing disparities. Future research should expand the range of variables considered, including household income and microbial analysis, to obtain a more comprehensive understanding of the factors influencing children’s oral health and inform the development of effective interventions.

## Data Availability

The data that support the findings of this study are available from the corresponding author upon reasonable request.

## Abbreviations

PFS: Pit and fissure sealant
FPM: First permanent molars
WHO: World Health Organisation
DMFT: decayed, missing, and filled teeth index
GDP: gross domestic product
LBW: low birth weight

## References

[1] Selwitz RH, Ismail AI, Pitts NB (2007). Dental caries. Lancet.

[2] Kassebaum NJ, Smith AGC, Bernabé E, Fleming TD, Reynolds AE, Vos T, Murray CJL, Marcenes W (2017). GBD 2015 oral health collaborators. Global, Regional, and National Prevalence, incidence, and disability-adjusted life years for oral conditions for 195 countries, 1990–2015: a systematic analysis for the Global Burden of Diseases, Injuries, and risk factors. J Dent Res.

[3] Wright JT (2018). The Burden and Management of Dental Caries in Older Children. Pediatr Clin North Am.

[4] Bernabe E, Marcenes W, Hernandez CR, Bailey J, Abreu LG, Alipour V, Amini S, Arabloo J, Arefi Z, Arora A, Ayanore MA, Bärnighausen TW, Bijani A, Cho DY, Chu DT, Crowe CS, Demoz GT, Demsie DG, DibajiForooshani ZS, Du M, El Tantawi M, Fischer F, Folayan MO, Futran ND, Geramo YCD, Haj-Mirzaian A, Hariyani N, Hasanzadeh A, Hassanipour S, Hay SI, Hole MK, Hostiuc S, Ilic MD, James SL, Kalhor R, Kemmer L, Keramati M, Khader YS, Kisa S, Kisa A, Koyanagi A, Lalloo R, Le Nguyen Q, London SD, Manohar ND, Massenburg BB, Mathur MR, Meles HG, Mestrovic T, Mohammadian-Hafshejani A, Mohammadpourhodki R, Mokdad AH, Morrison SD, Nazari J, Nguyen TH, Nguyen CT, Nixon MR, Olagunju TO, Pakshir K, Pathak M, Rabiee N, Rafiei A, Ramezanzadeh K, Rios-Blancas MJ, Roro EM, Sabour S, Samy AM, Sawhney M, Schwendicke F, Shaahmadi F, Shaikh MA, Stein C, Tovani-Palone MR, Tran BX, Unnikrishnan B, Vu GT, Vukovic A, Warouw TSS, Zaidi Z, Zhang ZJ, Kassebaum NJ, GBD 2017 Oral Disorders Collaborators (2020). Global, Regional, and national levels and Trends in Burden of oral conditions from 1990 to 2017: a systematic analysis for the global burden of Disease 2017 study. J Dent Res.

[5] Lu ZF, Zhang Y. Review of oral health status and Preventive Dentistry in Liaoning Province: 2005-2018.2020, Liaoning Science and Technology Press: Shenyang.

[6] W X. Fourth national oral health epidemiological survey report. People’s Medical Publishing House: Beijing; 2018.

[7] Lu HX, Tao DY, Lo ECM, Li R, Wang X, Tai BJ, Hu Y, Lin HC, Wang B, Si Y, Wang CX, Zheng SG, Liu XN, Rong WS, Wang WJ, Feng XP (2018). The 4th national oral health survey in the mainland of China: background and methodology. Chin J Dent Res.

[8] Zhu F, Chen Y, Yu Y, Xie Y, Zhu H, Wang H (2021). Caries prevalence of the first permanent molars in 6–8 years old children. PLoS ONE.

[9] Que L, Jia M, You Z, Jiang LC, Yang CG, Quaresma AAD, das Neves EMAA (2021). Prevalence of dental caries in the first permanent molar and associated risk factors among sixth-grade students in São Tomé Island. BMC Oral Health.

[10] Kashbour W, Gupta P, Worthington HV, Boyers D (2020). Pit and fissure sealants versus fluoride varnishes for preventing dental decay in the permanent teeth of children and adolescents. Cochrane Database Syst Rev.

[11] Office of the Ministry of Health, PRC. The guideline of the comprehensiveintervention program for oral diseases of children in the middle and westernregions (2011 Edition). Bull Ministry Health. 2011;7:44–61.

[12] World Health Organization (WHO). Oral Health Surveys: Basic Methods, World Health Organization, Geneva, 5th edition, 2013.

[13] Aldossary S, A Alamri M, A Alshiha A, A Hattan S, K Alfraih M, Alwayli YM. Prevalence of Dental Caries and Fissure Sealants in the First Permanent Molars among male children in Riyadh, Kingdom of Saudi Arabia. Int J Clin Pediatr Dent. 2018;11(5):365–70. 10.5005/jp-journals-10005-1541 PMC6379540 30787547

[14] Liu M, Xu X, Song Q, Zhang H, Zhang F, Lai G (2022). Caries prevalence of the first permanent molar and associated factors among second-grade students in Xiangyun of Yunnan, China: a cross-sectional study. Front Pediatr.

[15] Chen Z, Zhu J, Zhao J, Sun Z, Zhu B, Lu H, Zheng Y (2023). Dental caries status and its associated factors among schoolchildren aged 6–8 years in Hangzhou, China: a cross-sectional study. BMC Oral Health.

[16] Cheng YH, Liao Y, Chen DY, Wang Y, Wu Y (2019). Prevalence of dental caries and its association with body mass index among school-age children in Shenzhen, China. BMC Oral Health.

[17] Available from: http://www.stats.gov.cn [cited 2023.04.15].

[18] Schwendicke F, Dörfer CE, Schlattmann P, Foster Page L, Thomson WM, Paris S (2015). Socioeconomic inequality and caries: a systematic review and meta-analysis. J Dent Res.

[19] Costa SM, Martins CC, Pinto MQC, Vasconcelos M, Abreu MHNG (2018). Socioeconomic factors and caries in people between 19 and 60 years of age: an update of a systematic review and Meta-analysis of Observational Studies. Int J Environ Res Public Health.

[20] van der Tas JT, Kragt L, Elfrink MEC, Bertens LCM, Jaddoe VWV, Moll HA, Ongkosuwito EM, Wolvius EB (2017). Social inequalities and dental caries in six-year-old children from the Netherlands. J Dent.

[21] Tellez M, Gray SL, Gray S, Lim S, Ismail AI (2011). Sealants and dental caries: dentists’ perspectives on evidence-based recommendations. J Am Dent Assoc.

[22] Liu W, Xiong L, Li J, Guo C, Fan W, Huang S (2019). The anticaries effects of pit and fissure sealant in the first permanent molars of school-age children from Guangzhou: a population-based cohort study. BMC Oral Health.

[23] Ghaffari M, Rakhshanderou S, Ramezankhani A, Noroozi M, Armoon B (2018). Oral Health Education and Promotion Programmes: Meta-Analysis of 17-Year intervention. Int J Dent Hyg.

[24] Potisomporn P, Sukarawan W, Sriarj W (2019). Oral Health Education Improved oral health knowledge, attitudes, and Plaque Scores in Thai Third-grade students: a Randomised Clinical Trial. Oral Health Prev Dent.

[25] Gorbatova MA, Gorbatova LN, Pastbin MU, Grjibovski AM. Urban-rural differences in dental caries experience among 6-year-old children in the Russian north. Rural Remote Health. 2012;12:1999. Epub 2012 Jun 14. 22702845

[26] Christian B, Blinkhorn AS (2012). A review of dental caries in australian Aboriginal children: the health inequalities perspective. Rural Remote Health.

[27] Giacaman RA, Bustos IP, Bazán P, Mariño RJ (2018). Oral health disparities among adolescents from urban and rural communities of central Chile. Rural Remote Health.

[28] Koberova R, Radochova V, Zemankova J, Ryskova L, Broukal Z, Merglova V (2021). Evaluation of the risk factors of dental caries in children with very low birth weight and normal birth weight. BMC Oral Health.

[29] Cui Y, Chen D, Lin H, Tao Y. The association between low birth weight and/or preterm birth and dental caries -A systematic review and meta-analysis. Int J Dent Hyg. 2023;21(3):599–610. 10.1111/idh.12651 36524312

[30] Weng X, Lou Y, Tao R, Li Y, Cao D, Yu M, Ying B, Wang H (2021). The association between low birth weight and dental caries among 11-to-13-year-old school age children in Ningbo, China. BMC Pediatr.

[31] Chen L, Hong J, Xiong D, Zhang L, Li Y, Huang S, Hua F (2020). Are parents’ education levels associated with either their oral health knowledge or their children’s oral health behaviors? A survey of 8446 families in Wuhan. BMC Oral Health.

[32] Peres MA, Sheiham A, Liu P, Demarco FF, Silva AE, Assunção MC, Menezes AM, Barros FC, Peres KG (2016). Sugar Consumption and Changes in Dental Caries from Childhood to Adolescence. J Dent Res.

[33] van Loveren C (2019). Sugar Restriction for Caries Prevention: amount and frequency. Which Is More Important? Caries Res.

